# Effect of high-intensity laser therapy in patients with non-specific chronic neck pain: study protocol for a randomized controlled trial

**DOI:** 10.1186/s13063-023-07599-0

**Published:** 2023-08-31

**Authors:** Hernán Andrés de la Barra Ortiz, Mariana Arias Avila, Luis Gómez Miranda, Richard Eloin Liebano

**Affiliations:** 1 Exercise and Rehabilitation Sciences Institute, School of Physical Therapy, Faculty of Rehabilitation Sciences, Universidad Andres Bello, Santiago, 7591538 Chile; 2 Department of Physical Therapy, Federal University of São Carlos (UFSCar), Rodovia Washington Luis, km 235, São Carlos, São Paulo Brazil; 3 Physiotherapeutic Resources Research Laboratory, Department of Physical Therapy, Federal University of São Carlos (UFSCar), São Carlos, Brazil; 4 Department of Rehabilitation Sciences, University of Hartford, West Hartford, CT USA

**Keywords:** Lasers, Laser therapy, Chronic pain, Neck pain, Physical therapy modalities, Clinical trial protocol

## Abstract

**Background:**

Chronic non-specific neck pain (CNNP) is a prevalent musculoskeletal disorder known for its significant disability and economic burden, ranking second only to low back pain in musculoskeletal conditions. Physical therapy offers effective interventions for CNNP, including low-level laser therapy (LLLT). High-intensity laser therapy (HILT) is a recent treatment for musculoskeletal pain, but studies that support its use in CNNP are limited. The objective of this study is to assess the effect of high-intensity laser therapy on pain intensity in patients with CNNP, given the existing evidence on LLLT for this condition.

**Methods:**

This is a 2-arm, randomized, placebo-controlled trial with blinded evaluators. The research will be carried out in the laboratory of physical agents at the Andrés Bello University, Campus Casona de las Condes. Eligible participants include the entire internal and external community associated with Andrés Bello University suffering from chronic non-specific NP. Participants will be stratified by sex (4 subgroups) and randomized into 2 study groups: group 1 (HILT and stretching exercises) and group 2 (sham HILT and stretching exercises). Treatments will be performed twice a week for 4 weeks with 3 assessments: before treatment (T0), at the end of treatment (T1), and 12 weeks after treatment (follow-up) (T2). The main outcomes will be pain intensity at rest, pain intensity at movement (active cervical movements: flexion, extension, right and left side bending, and right and left rotation), and pain pressure threshold (average obtained for six evaluation points). Secondary outcome measures will include neck range of motion in the sagittal, coronal, and transverse planes and neck disability.

**Discussion:**

In this study, HILT’s effects on patients with non-specific NP will be compared to those of a sham laser intervention. This RCT will offer new evidence regarding the potential benefits of HILT in terms of pain intensity, range of movement, and disability in people suffering with non-specific NP.

**Trial registration:**

ClinicalTrials.gov NCT05689788. January 19, 2023.

## Introduction

### Background and rationale {6a}

Neck pain, a multifactorial musculoskeletal disorder, is the fourth leading cause of disability in adults, with a 30% annual prevalence [1, 2]. Individuals from developed countries, urban dwellers, and individuals engaged in office or computer work are more affected [3]. Neck pain is linked to sedentary habits, smoking, and psychological risk factors. Although it often resolves on its own or responds to treatment, 30 to 50% of patients may experience chronic pain [1, 2, 4, 5].

Neck pain is classified based on its duration (acute or chronic) and its mechanism (nociceptive, neuropathic, or nociplastic), depending on its association with the nervous system [1, 6]. Additionally, it is categorized as specific or non-specific based on its underlying cause [6]. Specific neck pain is associated to structural conditions such as radiculopathies, myelopathies, fractures, and joint inflammation. In contrast, chronic non-specific neck pain (CNNP) is often idiopathic and lacks identifiable structural injury [5, 6].

CNNP has a prevalence of around 50%, and its increasing occurrence among adolescents underscores the need for early intervention and preventive measures [4]. CNNP is associated with functional limitations, increased healthcare utilization, and frequent use of medication for pain relief [4]. As a public health concern, it leads to high socioeconomic costs, primarily due to job absenteeism. CNNP is linked to central sensitization, whose influence is important in conditions such as cervical myalgia or fibromyalgia [7]. CNNP can manifest as local or referred pain, hyperalgesia, reduced cervical mobility, and muscle tension. Additionally, cases of neuropathic pain may involve accompanying symptoms such as headaches, numbness, weakness, or tingling, with or without radiation to the upper limb [1, 3, 6]. CNNP medical treatments prioritize pain management using NSAIDs, opioids, or muscle relaxants, showing efficacy in acute NP cases [1, 8]. Other approaches include local injections of lidocaine or corticosteroids near nerve roots, zygapophysial joints, or cervical muscles, but evidence is inconclusive. Surgery is less common, reserved for radiculopathies or spinal cord compressions, with better short-term results compared to long-term [1, 8].

Laser therapy is widely used in physical therapy for tissue repair, wound healing, and pain reduction in musculoskeletal conditions [9, 10]. Its analgesic effects are associated with its ability to modulate the inflammatory process, release endogenous opioid peptides (β-endorphins), and decrease nerve conduction velocity, especially at higher potencies [9–12].

Laser radiation, present in the visible red and infrared light spectrum, varies in its biological penetration depth [12]. Laser production involves exciting atoms in a medium with free electrons, resulting in the emission of photons through stimulated radiation emission [11, 12]. These photons are absorbed by specific chromophores in tissues, such as water molecules, hemoglobin, melanin, and cytochrome c oxidase [11, 13, 14].

Laser therapeutic devices are categorized into two types: class IIIb, or low-level laser therapy (LLLT), and class IV, or high-intensity laser therapy (HILT), based on their emission power (less than or greater than 0.5 W, respectively) [15–17]. LLLT has athermal and superficial effects (3–4 cm) and is known for its photobiological effects (photobiomodulation) that stimulate or inhibit biological processes depending on the delivered energy dose (Arndt-Schultz law) [11]. In recent years, HILT has emerged as a novel approach for managing musculoskeletal pain, distinguished by its photothermal and photochemical effects [11, 13, 18]. With its higher power, HILT can deliver more energy to deep tissues, reaching greater depths owing to its infrared spectrum wavelengths (averaging depths of 10–12 cm) [18]. HILT offers distinct advantages over LLLT as it enables the delivery of higher energy over time. HILT allows for more energy deposition in deep tissues, resulting in both the biological effects of LLLT and thermal effects [13, 14, 18].

Currently, LLLT is widely recognized in physiotherapy as an effective resource for managing both acute and chronic neck pain and recommended by evidence-based guidelines [11, 15–17]. LLLT delivers immediate pain relief for acute neck pain and exhibits sustained effectiveness for up to 22 weeks post-treatment in chronic neck pain patients [10]. In contrast, HILT is being used for the management of musculoskeletal pain, reporting analgesic benefits and less disability in conditions such as osteoarthritis, epicondylalgia, and low back pain [15, 19–22]. Studies on the benefits of LLLT and HILT for musculoskeletal pain offer a basis for exploring class IV laser as a treatment for CNNP, building on the existing evidence on LLLT for this condition. However, there is limited evidence supporting HILT efficacy in the management of CNNP, with only a few studies available [6, 16].

### Objectives {7}

The main purpose of the research is to investigate whether pain intensity will decrease after HILT treatment compared with sham HILT after treatment and with a 3-month follow-up. Additionally, secondary outcomes such as changes in pain pressure threshold, active cervical range of motion (flexion, extension, left- and right-side bending, and left and right rotation), and cervical disability will be evaluated. The hypothesis of the study is that HILT will lead to greater pain intensity improvement than conventional treatment. The purpose of this article is to describe the methods and statistical analysis of this study so that this information can be made public.

### Trial design {8}

This is a 2-arm randomized placebo-controlled superiority trial (RCT) with patient and evaluator blinded to the group allocation. This study is reported in accordance with established clinical trial reporting standards: the Consolidated Standards of Trial Reporting (CONSORT). The protocol was developed following the recommendations of Standard Protocol Elements: Recommendations for Interventional Trials (SPIRIT).

The study has been approved by the Ethics Committee of the Eastern Metropolitan Health Service (SSMO), Santiago de Chile, following the Helsinki principles (approval date: October 26, 2022. N° 20200234) [23].

## Methods: participants, interventions, and outcomes

### Study setting {9}

The research will be carried out in the physical agent’s research and intervention laboratory of the Physical Therapy Career program at Andrés Bello University, Santiago de Chile. The protocol of this study has been registered in the Clinical Trials platform (http://clinicaltrials.gov), a resource provided by the US National Library of Medicine (ClinicalTrials.gov Identifier: NCT05689788).

### Eligibility criteria {10}

Participants in this study will be people of the internal and external communities associated with Andrés Bello University. Participants will be recruited through mailings and posters and will be contacted via email or telephone to schedule an in-person meeting at the laboratory. The evaluators will verify whether the patients will be eligible to participate in the study based on patient history and clinical examination.

The inclusion criteria are as follows:

- Participants must be at least 18 years old.
- Participants of both sexes.
- CNNP, defined as pain or discomfort in the cervical region between the superior nuchal line and the spinous process of T1 or the shoulder girdle, with the following criteria: NP in the last 3 months or more; a questionnaire score of cervical disability (NDI) equal to or greater than 5; and NP at rest of 3 or greater on a 0 to 10 numerical pain rating scale (NPRS) [3, 24].

The exclusion criteria are as follows [11]:

- Neck or shoulder musculoskeletal injuries in the last 3 months (fractures, sprains, tendinopathies, dislocations, or muscle tears).
- Osteosynthesis materials close to the shoulders, neck, or surrounding areas.
- Wounds or skin changes in the shoulder and/or neck region (such as psoriasis, scars, or burns).
- Analgesic, anti-inflammatory, or muscle relaxant drugs (either for continuous use or those used during the study).
- Neurological alterations such as paresthesia, loss of sensation (partial or complete), decrease in strength, and color changes in the neck, arms, forearms, or hands.
- Photosensitivity diagnosis.
- Skin phototypes V and VI (Fitzpatrick scale).
- Presence of solar urticaria or adverse reactions to sunlight.
- Presence of dermatomyositis, systemic lupus erythematosus, hepatic porphyria, cutaneous carcinoid syndrome, or pellagra.
- Cancer or tumors of some type have been diagnosed in the last 5 years.
- Epilepsy.

### Who will take informed consent? {26a}

All patients included in the study must validate their participation by signing an informed consent with the evaluator’s prior explanation.

### Additional consent provisions for collection and use of participant data and biological specimens {26b}

N/A. Not applicable for this study.

## Interventions

### Explanation for the choice of comparators {6b}

HILT’s studies on CNNP are limited and done without using a placebo control. In this study, HILT will be compared with a sham HILT application. Placebo comparators are considered the gold standard for evaluating the efficacy of an intervention in clinical trials. This will allow assessing the analgesic efficacy of HILT as well as its effect on other variables like cervical ROM and neck disability.

### Intervention description {11a}

Participants will be divided into two main groups: group 1 (HILT) and group 2 (sham HILT). Within each group, there will be two subgroups for men and women, determined through a simple randomization process and stratification by sex, utilizing the research randomizer program [25]. his allows the researchers to analyze the outcomes of interest by group and by subgroup. This study recognizes the superior benefits of passive stretching for flexibility improvement, post-exercise recovery, and range of motion limitations, especially in CNNP patients [26–29]. Both groups will receive as a base treatment a bilateral passive stretching exercise plan of the upper trapezius muscle, levator scapulae, and scalenes in 3 series for 30 s [27, 28]. Treatments will be performed twice a week for 4 weeks with 3 assessments: before treatment (T0), at the end of treatment (T1), and 12 weeks after treatment (follow-up) (T2).

#### High-intensity laser application (HILT) — experimental group

**Fig. 1:**
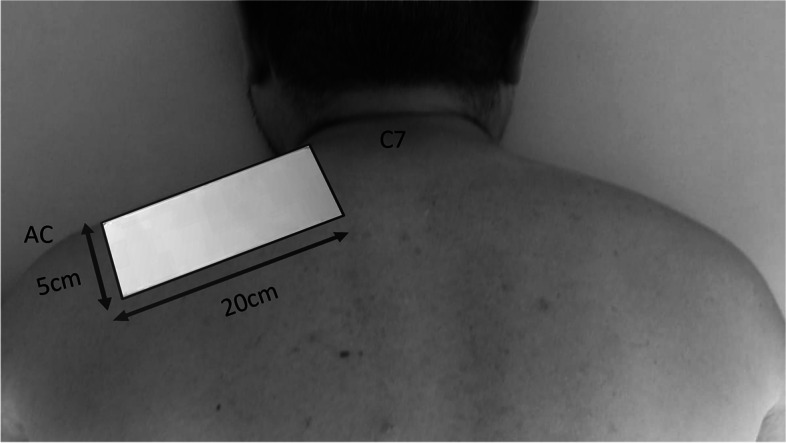
Delimitation of the trapezius treatment area

HILT will be applied on the map of 12 points evaluated with algometry using the spot technique, while the upper trapezius muscle belly will be treated with a scanning technique, covering an area of 100 cm^2^ [24, 30]. The perimeter of the trapezium treatment area will be delimited using a 5 × 20-cm transparent lens that will be placed in the center of the area limited by the spinous process of C7 and the acromion (Fig. 1).

For the laser therapy application, the 12W BTL-6000 equipment will be used. The energy dose proposed by Dundar et al. that combines the HILT scanning and spot techniques [30] is as follows: An energy of 10 J will be delivered per point (60 J in total for each side), and 1000 J of manual scanning for 100 cm^2^ of the upper portion will be divided into two phases of 500 J (a total of 1000 J for each side), delivering a total of 1060 J for each side of the body in each treatment. Table 1 shows the technical specifications of the laser equipment and the treatment parameters. The treatment protocol will be carried out in three phases:

- *Phase 1 (scanning)*: continuous mode, peak power of 12 W for 42 s, delivering a total energy of 500 J in the upper trapezius muscle belly (area of 100 cm^2^).
- *Phase 2 (spot technique)*: pulsed mode, duty cycle of 25%, average power of 1 W for 10 s, delivering 10 J per point, completing a total of 60 J for each side.
- *Phase 3 (scanning)*: continuous mode, peak power of 6 W for 83 s, delivering a total energy of 500 J in the upper trapezius muscle belly (area of 100 cm^2^).

**Table 1:** High-intensity laser therapy characteristics and parameters

**Laser technique specifications**	
Wavelength	1.064 nm
Output power	12 W
Beam divergency	35°
Emission mode	Continuous or pulsed
Frequency (only for pulsed mode)	1–100 Hz (fixed duty cycle 25%)
Spacer	30 mm
Spot size	3.14 cm^2^
**Laser treatment parameters for each side**	
Peak power (W)	Phase 1: 12 W Phase 2: 4 W Phase 3: 6 W
Emission mode	Phase 1: continuous mode (duty cycle 100%) Phase 2: pulsed mode at 100 Hz (duty cycle 25%) Phase 3: continuous mode (duty cycle 100%)
Mean power (W)	Phase 1: 12 W Phase 2: 1 W per point Phase 3: 6 W
Application technique	Phase 1: contact, manual scan for 100 cm^2^ Phase 2: contact, punctual technique for 6 points per side Phase 3: contact, manual scan for 100 cm^2^
Application angle	90°, perpendicular to the skin
Power density (W/cm^2^)	Phase 1: 0.038 W/cm^2^ Phase 2: 0.31 W/cm^2^ Phase 3: 0.019 W/cm^2^
Treatment time (sec)	Phase 1: 42 s Phase 2: 10 s per point (60 s for 6 points) Phase 3: 83 s
Energy density (J/cm^2^)	Phase 1: 5 J/ cm^2^ Phase 2: 3.1 J/ cm^2^ per point Phase 3: 5 J/c cm^2^
Energy delivered per side (J)	Phase 1: 500 J Phase 2: 60 J Phase 3: 500 J Total per side = 1.060 J

*Abbreviations: cm*^*2*^ square centimeters, *Hz* hertz (cycles per second), *J* joules, *mm* millimeters, *nm* nanometers, *sec* seconds, *W* watts

#### Sham high-intensity laser application (HILT) — control group

For sham HILT intervention, individuals will be treated with the same care as the current intervention group, including dose, therapy time, individual care, and the physical space where the therapy will be performed. The placebo intervention will be performed as a control.

#### Stretching exercises — experimental and control groups

While both active and passive stretching can enhance flexibility, passive stretching tends to yield greater improvements, making it recommended for addressing range of motion restrictions in CNNP [26–29]. As a result, both groups in the study will be subjected to a bilateral passive stretching protocol targeting the upper trapezius, levator scapulae, and scalenes muscles.

The practitioner will stretch the participants’ muscles by taking the neck to the points of maximum tension and holding that position. The stretches will consist of 3 series of 30 s, with an interval of 30 s between series [27, 28]. The exercises will be carried out with the participant in a seated position in a chair with a backrest. Stretching exercises will be performed by a physical therapist after the treatment assigned to each group (HILT or sham HILT).

### Criteria for discontinuing or modifying allocated interventions {11b}

There will be no changes in assignments, nor will migration of individuals between groups be allowed. If people discontinue treatment, the analysis will be by “intention to treat,” recording the reasons for leaving the study.

### Strategies to improve adherence to interventions {11c}

To minimize data loss, all participants will be provided with guidance when they sign the informed consent form and commit to attending on the scheduled treatment dates. Participants will receive an appointment card to attend sessions. An evaluator will be responsible for notifying and monitoring the participants on a weekly basis (via telephone contact, WhatsApp, and/or email) and accompanying them during the research.

### Relevant concomitant care permitted or prohibited during the trial {11d}

During the experiment, concurrent treatments such as chronic diabetes, hypertension, anxiolytics, and antidepressant drugs will be accepted. Throughout the course of the trial, participants will not be allowed to start any other interventions or medications, especially on the days that the evaluations and treatments are carried out.

### Provisions for post-trial care {30}

During the initial evaluation session, participants will be given a document outlining all the considerations that should be made during the study period. The principal researcher will provide participants with his phone number, the researchers’ email addresses, and contact information about the physical therapy department at Andrés Bello University. The evaluators will keep an electronic record (in Microsoft Excel) of the medications taken during the week. In general, the treatments are safe and do not pose risks for the participants. However, if the individual exhibits any negative effects, they will be referred to the university health center for evaluation and treatment.

### Outcomes {12}

**Fig. 2:**
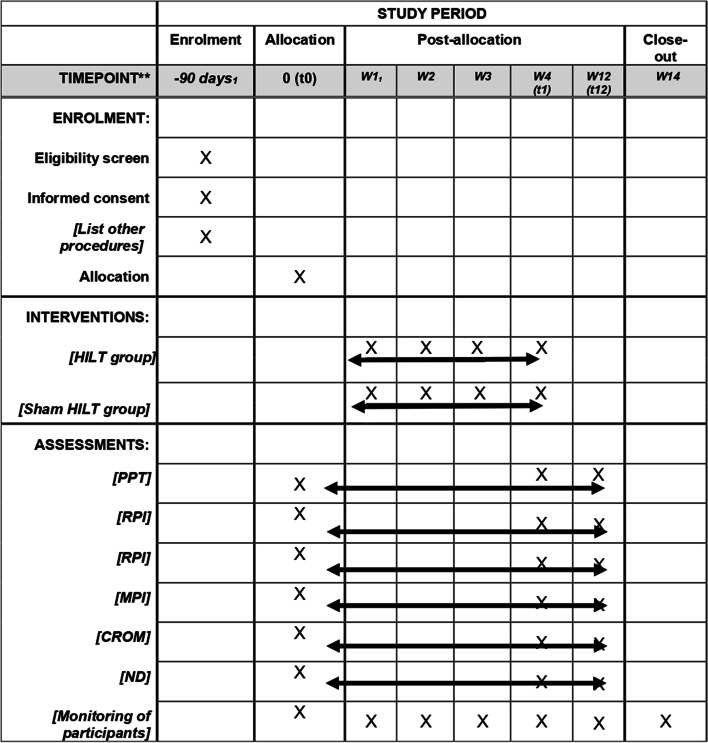
Schedule of enrolment, interventions, and assessments. Abbreviations: CROM, cervical range of motion; HILT, high-intensity laser therapy; MPI, pain intensity at movement; ND, neck disability; PPT, pain pressure threshold; ROM, range of motion; RPI, pain intensity at rest; t1, post-treatment evaluation; t2, follow-up evaluation; W, week

**Fig. 3:**
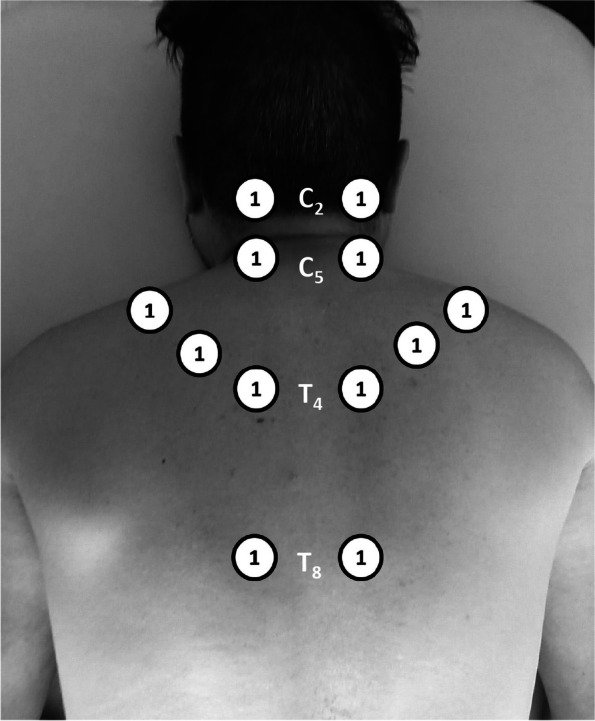
PPT recording points

The main outcomes will be the changes in pain intensity at rest (RPI), pain intensity at movement when performing active cervical movements in the sagittal (flexion–extension), coronal planes (neck right and left side bending) and transverse plane (right and left rotation) (MPI), and pressure pain threshold obtained as a mean of six points of the cervical spine and shoulder region according to the protocol of Rampazo et al. (Fig. 3) (PPT). Secondary outcome measures, on the other hand, will include differences in cervical range of motion (CROM) and neck disability (ND) after the HILT application in participants with chronic nonspecific NP. The evaluations will be carried out by two independent evaluators: evaluator 1 (PPT and PI) and evaluator 2 (CROM and ND). PPT will be evaluated with algometry at six established bilateral points [24] (Fig. 3), RPI and MPI with numeric pain rating scale (NPRS) [31], cervical ROM with an inclinometer (CROM device) [32–34], and neck disability (ND) through the neck disability index (NDI) [35, 36]. The evaluations of the outcome measures of interest will be carried out in three instances: pretreatment (T0: baseline), the fourth week (T2: the 8th HILT session, end of treatment), and week 20 (T3: 12 weeks post-treatment or follow-up) (Fig. 2).

#### Pressure pain threshold (PPT)

The evaluation of the PPT will be carried out through pressure algometry. The procedure will be performed with the participant in the prone position with the arms at the side of the trunk. For the study, the digital pressure algometer FPX (Wagner) will be used. Six points of the cervical spine and shoulder region will be evaluated according to the protocol of Rampazo et al. (Fig. 3) [24]: 2 cm lateral to the spinous processes of C2, C5, T4, and T8, midpoint of the upper trapezius muscle (between the spinous process of C7 and the acromion) and levator scapula muscle (2 cm superior to the superior angle of the scapula). Kilograms of pressure per square centimeter of surface (kg/cm^2^) will be recorded. The measurements will be carried out three times for each point with an interval of 30 s, and the mean value will be recorded as the pressure pain threshold (PPT). The kg/cm^2^ of pressure at which the participant reported pain with the test will be recorded in a Microsoft Excel® spreadsheet [24, 37, 38].

Intra-rater reliability for the PPT measurement will be determined with the intra-class coefficient after assessing the PPT of the midpoint of the upper trapezius muscle belly in 13 healthy volunteers not involved in the study with a 48-h interval between assessments [39].

#### Pain intensity at rest and on movement (RPI and MPI)

Pain intensity will be evaluated through the numerical pain scale (NPRS: intra-rater reliability; intraclass correlation coefficient, ICC = 0.76 (95% CI: 0.51,0.87) [29]. The RPI will equal the magnitude of neck pain reported by the participants at rest, while the MPI will be represented as the magnitude of pain reported by the participants when performing active cervical movements in the sagittal (flexion–extension), coronal (neck right and left side bending), and transverse planes (right and left rotation) [31]. The paint intensity with NPRS will be assessed for each movement using a single attempt (a total of six movements). The RPI and MPI exams will be performed by the participant in a seated position, keeping the back straight and supported on a backrest. The RPI and MPI values will be recorded on a Microsoft Excel® spreadsheet.

#### Cervical range of motion (CFROM) — CROM device

The CROM will be equivalent to the range of active movement of the head with respect to the trunk in the movements of flexion, extension, right and left side bending, and left and right rotation [32–34]. The ranges of movement will be evaluated through an inclinometer (CROM device), registering the degrees of movement for the described movements (inter-rater reliability: ICC extension = 0.98 (95% CI 0.95, 0.99); ICC flexion = 0.89 (95% CI 0.73,0.96); ICC left rotation = 0.95 (95% CI 0.87, 0.98); and ICC right rotation = 0.92)) [32–34]. The CROM values for each movement will be recorded. Three active CROM attempts will be made (30 s between each attempt), recording the best value for each movement.

- *Cervical flexion and extension (sagittal plane)*: The participant will be sitting with a straight back to stabilize the thoracic spine. The physical therapist will place the inclinometer in the sagittal plane over the upper aspect of the head (midline), stretching the skin of the skull to decrease movement of the skin during the measurement, while using the other arm to help stabilize her trunk. The inclinometer will place at 0 to subsequently request maximum flexion and extension movements.
- *Cervical side bending assessment (coronal plane)*: The participant will be sitting with a straight back to stabilize the thoracic spine or the wall to the side to stabilize the trunk. The therapist places the inclinometer on the head in the frontal plane, spreading the skin on the scalp as described above. The inclinometer will be set to zero, and the participant will be asked to tilt their neck to one side while the angle is recorded. The procedure is then repeated on the other side.
- *Cervical rotation (horizontal plane)*: The participant will be placed in the supine position. The physiotherapist will place the inclinometer on the model’s forehead and set the inclinometer to 0. The participant is then asked to rotate the head maximally to the right, and the measurement is recorded. The same process was repeated to measure the rotation to the left.

#### The Neck Disability Index

Neck disability will be considered any partial or total limitation of the functionality of the cervical region due to pain that makes the normal development of the person’s activities of daily living impossible or difficult. Neck disability will be assessed through the Neck Disability Index (NDI), which consists of 10 sections with questions related to symptoms and activities of life that may be limited by NP and has been validated in Spanish [35]. Each section consists of questions scored from 0 to 5, with greater disability being associated with a higher score (NDI, inter-rater reliability: ICC = 0.93 (95% CI 0.86, 0.97) [35, 36]. The neck disabilities percentages will be recorded.

### Participant timeline {13}

**Fig. 4:**
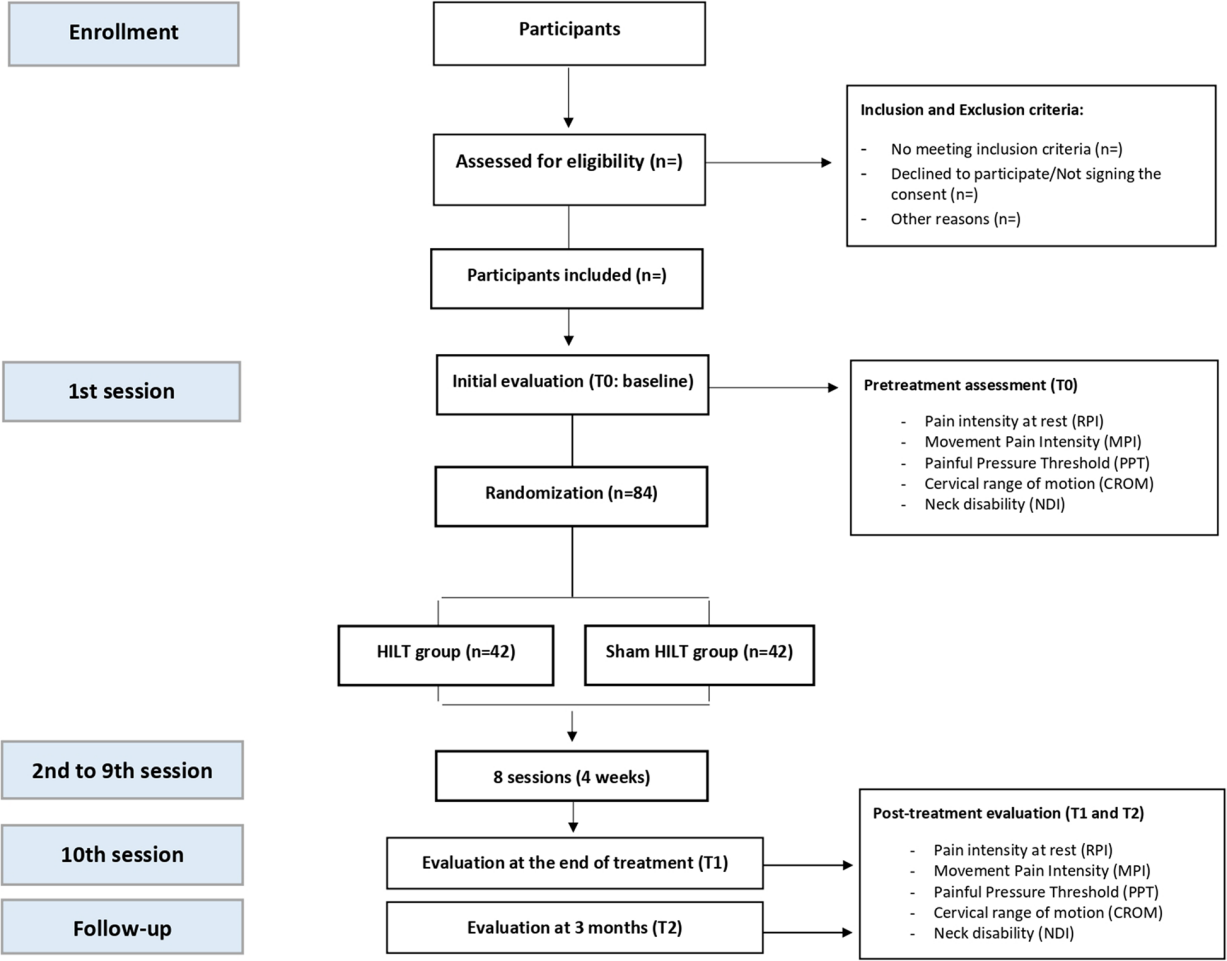
Flowchart of the randomized clinical trial

Figure 4 displays the participant timeline.

### Sample size {14}

The sample size was determined with the G-Power program using a power of 0.80 (1-*β*), a reliability of 95%, an error of 5% (*α*), and an effect size of *d* = 0.6 (Cohen’s *d*) with reference to previous studies that determined differences in mean pain intensity between experimental and control groups after HILT treatment with effect sizes of 0.53 [30, 40]. Based on the above, the calculated sample size is 72 subjects, with at least 36 subjects per group. The study will include 84 participants (42 per group) in recognition of the crucial role of sample size in determining the power and impact of the study. An additional 15% of participants were considered for possible losses during the follow-up (bias due to abandonment). This is consistent with what the literature recommends, which suggests including at least 10% more participants, and with the PEDro scale (criterion 8), which advocates for analyzing at least 85% of the data to maintain data validity and obtain more robust statistical results and analysis [41, 42].

### Recruitment {15}

The study’s dissemination will take place through Andrés Bello University’s official channels (mailing and publication on the institutional website). Additionally, the Physical Therapy’s School communication channels will be utilized (through mailings, social networks, and posters). Volunteers will be contacted by phone or email to attend the research lab.

## Assignment of interventions: allocation

### Sequence generation {16a}

The selected participants will be allocated into two groups, group 1 (HILT) and group 2 (sham HILT), using a simple randomization process via the website research randomizer [25]. A block randomization with a 1:1:1:1 allocation will be performed, and participants will be stratified by sex, resulting in four subgroups with equal numbers of women and men per group (*n* = 40 per group, 20 men, and 20 women). Each participant will have an equal probability of being randomly allocated to either group 1 or group 2.

### Concealment mechanism {16b}

After the website generates a numerical sequence (https://www.randomizer.org), a concealed allocation will be performed in consecutively numbered opaque envelopes. The envelopes will be sealed, and they will be stored in a secure cabinet. Sample randomization and concealed allocation will be carried out by the principal researcher. The researcher in charge of administering the treatments will open the envelopes just prior to the intervention.

### Implementation {16c}

Four independent physical therapists will work on this study, and each of them will have a specific role. The principal researcher (R1) will oversee participant recruitment, participant registration, randomization, and the random allocation sequence. Moreover, the interventions will be implemented by a researcher (R2) (HILT, sham HILT, and exercise). All evaluations will be performed by a third researcher (R3) (RPI, MPI, PPT, CROM, and ND). The principal researcher will examine and record the data.

## Assignment of interventions: blinding

### Who will be blinded {17a}

Evaluators will be blinded to the randomization and intervention processes; they will be responsible only for the evaluation procedures and will not receive information about the assignment of individuals to groups. Participants and the principal researcher responsible for the treatment will not be blinded due to the nature of the interventions.

### Procedure for unblinding if needed {17b}

The evaluators will not be allowed to unblock the blinding.

## Data collection and management

### Plans for assessment and collection of outcomes {18a}

All the data will be collected weekly, saved in a Microsoft Excel spreadsheet, and stored on the institutional Portal Office 365 cloud (Andrés Bello University, UNAB). During the development of this research, the data will be restricted and for the exclusive use of registered researchers and participants. All confidential participant data will not be available to the public.

### Plans to promote participant retention and complete follow-up {18b}

Principal researcher (R1) and an evaluator (R2) will be responsible for notifying and monitoring the participants on a weekly basis (via telephone contact, WhatsApp, and/or email) and accompanying them during the research.

### Data management {19}

The principal researcher (R1) will be responsible for the data management.

### Confidentiality {27}

All the information generated during the study will be anonymized, that is, coded without revealing personal data, and handled confidentially. Only the principal researcher will have access to it, and he will keep all the data safe. The data will be stored on the institutional platform (Andrés Bello University), which will be accessible via the institutional Office 365 cloud. Only researchers from the UNAB who participate in this research will have the authorization to access these stored documents.

### Plans for collection, laboratory evaluation, and storage of biological specimens for genetic or molecular analysis in this trial/future use {33}

N/A. Not applicable to this clinical trial.

## Statistical methods

### Statistical methods for primary and secondary outcomes {20a}

The descriptive statistics for the variables RPI, MPI, PPT, CROM, and ND will be used as analysis measures: averages and standard deviation (*x*, SD), median and interquartile range (mean, IQR), depending on the distribution of the data (test of Kolmogorov–Smirnov normality). From these data, a table will be constructed with the demographic data of the participants by group. The secondary variables sex and body mass index (BMI) will be presented with frequencies and averages or medians, respectively, according to the analysis of the normality of the data. For the inferential statistical analysis of the outcome measures, the Kolmogorov–Smirnov normality test and the Bartlet test will be used to determine the distribution of the variables PPT, RPI, MPI, CROM, and ND, and the corresponding homoscedasticity of the variances obtained for these variables in the evaluations (T0, T1, and T2). According to the results, parametric or non-parametric tests will be selected: the changes in the measurement variables within and between groups will be analyzed with the two-factor ANOVA or the Kruskal–Wallis test, depending on the distribution of the data. Subsequently, a post hoc analysis will be carried out with the Tukey or Bonferroni test, depending on the statistical differences. The significance level for all statistical tests will be set at 0.05. Data will be analyzed using IBM Statistical Package for the Social Sciences (SPSS) software (version 26; SPSS Inc.; Chicago, IL) by an investigator who is blind to group assignment.

### Interim analyses {21b}

No provisional analyses will be performed. In the event of participant discontinuity, the statistical analysis will employ intention-to-treat principles to ensure a comprehensive and unbiased evaluation of the data.

### Methods for additional analyses (e.g., subgroup analyses) {20b}

A subgroup analysis will be carried out according to sex.

### Methods in analysis to handle protocol non-adherence and any statistical methods to handle missing data {20c}

In cases of discontinuity, the missing data will be treated according to the principle of “intention to treat” to perform an inferential statistical analysis.

### Plans to give access to the full protocol, participant-level data and statistical code {31c}

N/A. Not applicable to this study.

## Oversight and monitoring

### Composition of the coordinating center and trial steering committee {5d}

N/A. Not applicable to this research.

### Composition of the data monitoring committee, its role and reporting structure {21a}

The principal researcher will be responsible for recording and organizing the data obtained in this investigation that will come from the documents and electronic records: informed consent, demographic data, clinical characteristics of the participating individuals in the research, PPT values, RPI and MPI values, cervical range of motion values, and a disability score. The metadata will be descriptive and administrative, derived from data collection, and available in the digital repository of the university library.

### Adverse event reporting and harms {22}

In general, there are minimal risks because the study will be carried out under strict safety regulations, following the recommendations of the literature and biosafety for the application of laser therapy. The exclusion criteria will make it possible to eliminate any participant with complications from phototherapy. Any harm or complication related to the treatments will be reported to the Eastern Metropolitan Health Service ethics committee for Human Research (SSMO). The same applies to possible ethical issues that may arise during the research.

### Frequency and plans for auditing trial conduct {23}

Not applicable to this research.

### Plans for communicating important protocol amendments to relevant parties (e.g. trial participants, ethical committees) {25}

The SSMO Ethics Committee approved this protocol, and it was registered in the Clinical Trials Platform (https://clinicaltrials.gov) (NCT05689788). All amendments and adjustments proposed by the ethics committee were sent to the ethics committee before study approval (October 26, 2022. N° 20200234).

### Dissemination plans {31a}

Once the study is finished, the data will be registered in the repository of the Library Center of the Andrés Bello University. The results of this study will be published in a scientific journal.

## Discussion

The World Association for Laser Therapy (WALT) has proposed dosing recommendations for LLLT therapy in various musculoskeletal conditions; however, no recommendations for HILT have been documented [15, 20, 43]. HILT is a recent resource that has been incorporated into physical therapy for the management of musculoskeletal pain. Trials are emerging suggesting that HILT could decrease pain in conditions such as rheumatoid arthritis, osteoarthritis, low back pain, and NP, but the number and quality of studies are limited. An advantage of HILT over low-intensity lasers (class IIIb) is that it combines the photobiomodulation effects with those of deep thermotherapy, reaching greater depths for its wavelength [15, 20].

This randomized controlled trial will investigate the effect of HILT in patients with non-specific chronic NP and contribute new evidence that is currently limited. This study has a high-quality design that results in high-quality evidence that can be used to compare the analgesic efficacy and effectiveness of HILT to a placebo. In addition, effects can be evaluated if their effects transcend improvements in the range of movement and improved functionality.

The HILT group is expected to obtain significant analgesic differences in terms of a higher pain pressure threshold and lower pain intensity during movement, as well as a greater cervical range of motion and less disability at the end of treatment compared to the control group (sham HILT).

## Trial status

The protocol was approved on October 26, 2022, by the Eastern Metropolitan Health Service ethics committee for Human Research of Santiago (Chile). The initial recruitment date will be April 30, 2023, and the approximate date of completion of the recruitment of participants will be the second half of 2024.

## Data Availability

N/A. Not applicable.

## Abbreviations

CROM: Cervical range of motion
HILT: High-intensity laser therapy
LLLT: Low-level laser therapy
MPI: Pain intensity at movement
ND: Neck disability
NTC: Clinical trial number (clinical trials register)
NDI: The Neck Disability Index
PI: Pain intensity
PPT: Pain pressure threshold
ROM: Range of motion
RPI: Pain intensity at rest
SSMO: Ethics Committee of East Health Service
UNAB: Andres Bello University
VAS: Visual analog scale
